# A multidisciplinary design tool for robotic systems involved in sampling operations on planetary bodies

**DOI:** 10.1007/s12567-020-00330-8

**Published:** 2020-08-17

**Authors:** Dario Riccobono, Giancarlo Genta, Scott Moreland, Paul Backes

**Affiliations:** 1 Department of Mechanical and Aerospace Engineering (DIMEAS), Politecnico Di Torino, Corso Duca degli Abruzzi 24, 10129 Turin, Italy; 2 Jet Propulsion Laboratory, California Institute of Technology, 4800 Oak Grove Drive, Pasadena, CA 91109 USA

**Keywords:** Design tool, Sampling, Robotic systems, Lander, Planetary exploration

## Abstract

The analysis of robotic systems (e.g. landers and rovers) involved in sampling operations on planetary bodies is crucial to ensure mission success, since those operations generate forces that could affect the stability of the robotic system. This paper presents MISTRAL (MultIdisciplinary deSign Tool for Robotic sAmpLing), a novel tool conceived for trade space exploration during early conceptual and preliminary design phases, where a rapid and broad evaluation is required for a very high number of configurations and boundary conditions. The tool rapidly determines the preliminary design envelope of a sampling apparatus to guarantee the stability condition of the whole robotic system. The tool implements a three-dimensional analytical model capable to reproduce several scenarios, being able to accept various input parameters, including the physical and geometrical characteristics of the robotic system, the properties related to the environment and the characteristics related to the sampling system. This feature can be exploited to infer multidisciplinary high-level requirements concerning several other elements of the investigated system, such as robotic arms and footpads. The presented research focuses on the application of MISTRAL to landers. The structure of the tool and the analysis model are presented. Results from the application of the tool to real mission data from NASA’s Phoenix Mars lander are included. Moreover, the tool was adopted for the definition of the high-level requirements of the lander for a potential future mission to the surface of Saturn’s moon Enceladus, currently under investigation at NASA Jet Propulsion Laboratory. This case study was included to demonstrate the tool’s capabilities. MISTRAL represents a comprehensive, versatile, and powerful tool providing guidelines for cognizant decisions in the early and most crucial stages of the design of robotic systems involved in sampling operations on planetary bodies.

## Introduction

Robotic systems such as landers and rovers have been a crucial component for the exploration of planetary bodies since the beginning of the space exploration history. The Soviet Union’s lander Luna 9 was the first human-made object performing a soft landing on the surface of a planetary body (i.e. the Moon) in 1966. Since then, fleets of robotic systems have explored the Solar System, touching down on several planetary bodies, and pushing the limits of our knowledge of Solar System.

Robotic exploration began with several missions to the Moon continuing to the present day, from the US and Soviet Union’s probes of the 1960s and 1970s to the more recent Chinese missions Chang’e 3 and its follow-up Chang’e 4 [1, 2]. In the inner Solar System, several of the Soviet Union’s lander missions visited Venus in the 1970s and 1980s, such as the probes of the Venera and Vega programs. A notable case is represented by the US Pioneer Venus Multiprobe that delivered one large and three smaller probes to the surface of Venus. One of the probes survived on the surface for over one hour. Among planetary bodies, Mars was a historic privileged target of space exploration with several missions including orbiters, rovers and landers. In 1971, Soviet Union’s probes Mars 2 and Mars 3 were the first probes achieving the surface of Mars, both carrying a small tethered rover. Because of some malfunctions, they were not able of returning useful data. In 1976, the US landers Viking 1 and 2 were the first probes returning useful data from the surface of Mars [3], followed by the US landers Mars Pathfinder in 1997 [4] and Phoenix in 2008 [5]. The US InSight lander, which touched down in late 2018, is the most recent of a long series of Mars lander missions [6]. In the outer Solar System, Saturn’s moon Titan was the only planetary body of the outer Solar System visited by a landing mission to date. The European lander Huygens, part of the Cassini-Huygens mission, touched down on Titan in 2005 [7]. About small Solar System bodies, various landing missions also visited comets and asteroids. In 2001, the NEAR spacecraft performed the first landing on a small Solar System body, asteroid 433 Eros. The European mission Rosetta first delivered a lander, named Philae, on the surface of the comet 67P/Churyumov-Gerasimenko in 2014 [8]. On the other hand, MASCOT and MINERVA-II were the first landers/hoppers landing on asteroid Ryugu as part of the Japanese mission Hayabusa 2 in late 2018 [9].

Proposed future missions include significant robotic contributions for planetary science in either the inner and the outer Solar System, including planetary bodies such as Mars, comets, asteroids and ocean worlds [10]. Among ocean worlds, Saturn’s moon Enceladus and Jupiter’s moon Europa are the target of several mission proposals with the aim of finding potential life traces [11, 12].

Table 1 provides an overview of the main robotic lander configurations adopted in space exploration. The overview includes information on the configuration of legs and body and the Robotic Arm (RA), manipulator or deployable that perform the mission tasks.

**Table 1:** Overview of the main robotic lander configurations adopted in space exploration

Lander name	Landing configuration	Body configuration	RA configuration	Notes	References
Mars Pathfinder	1 point of contact with the surface arranged as a flat landing platform	Non-regular hexagon	Not applicable	The lander carried a small rover on the surface	[13]
Beagle-2	1 point of contact with the surface arranged as a flat landing platform	Shallow bowl	4 rotational DOFs	The RA was designed to perform scientific instrument and sampling system deployment to the surface	[14]
Venera 13, 14	1 point of contact with the surface arranged as a ring-shaped landing platform	Cylinder	1 rotational DOF	The RA performed scientific instrument deployment to the surface. The lander was equipped with a sampling system that operated at its location, fixed to the lander’s body	[15]
Surveyor 3, 4, 5, 6, 7	3 points of contact with the surface arranged as a regular triangle shape	Regular triangle	1 linear and 2 rotational DOFs	On Surveyor 3, 4 and 7, the RA performed sampling system deployment to the surface and supported sampling operations On Surveyor 5 and 6, the RA performed scientific instrument deployment to the surface	[16–20]
Viking 1, 2	3 points of contact with the surface arranged as a regular triangle shape	Non-regular hexagon	1 linear and 2 rotational DOFs	The RA performed sampling system deployment to the surface, supported sampling operations and delivered the sample collected to the scientific instrument	[3, 21, 22]
Mars Polar lander	3 points of contact with the surface arranged as a regular triangle shape	Regular hexagon	4 rotational DOFs	The RA was designed to perform sampling system deployment to the surface, to support sampling operations and deliver the sample collected to the scientific instrument	[23]
Phoenix	3 points of contact with the surface arranged as a regular triangle shape	Regular hexagon	4 rotational DOFs	The RA performed sampling system deployment to the surface, supported sampling operations and delivered the sample collected to the scientific instrument	[5]
Philae	3 points of contact with the surface arranged as a regular triangle shape	Non-regular hexagon	Not applicable	The lander was equipped with a sampling system that was designed to operate at its location, fixed to the lander’s body. The lander body had a 1 rotational DOF about the vertical, central joint of the 3-leg landing gear	[24]
InSight	3 points of contact with the surface arranged as a regular triangle shape	Regular hexagon	4 rotational DOFs	The RA performs scientific instrument deployment to the surface	[6]
Luna 16, 20, 24	4 points of contact with the surface arranged as a rectangle shape	Cylinder	1 linear DOF	The RA performed sampling system deployment to the surface and supported sampling operations	[25–27]

Since the beginning of space exploration, robotic systems (e.g. landers and rovers) were used for performing surface imaging and collecting data about the environment by using scientific instruments. As a result, surface and subsurface sampling and sample collection soon became one of the primary goals of robotic missions, mainly for in-situ analysis but also for sample return to Earth. Sampling operations can be performed by using several tools such as scoops, drills, backhoes, etc. Such tools might be fixed to the lander or supported and carried around by a RA in several configurations, as shown in Table 1. The RA configuration is selected to fulfill mission requirements (e.g. performing sampling or sensing at different locations on the surface). The RA has the purpose of placing the sampling tool in the desired spot on the surface while providing, together with the whole robotic system, reaction against the forces generated during the sampling operation. To guarantee a nominal sampling operation, crucial to mission success, it is required that the forces generated by the sampling system do not affect the stability of the whole system. The traditional approach for investigating various concepts for a robotic mission involved in sampling operations relies on the preliminary definition of a set of potential sampling tool candidates [28]. Therefore, ad-hoc analyses on the stability of the whole robotic system need to be performed every time a design parameter (e.g. physical and geometrical characteristics of the robotic system, properties related to the environment, physical and geometrical features of the sampling tool) is modified [29–31]. At a higher level, the same process must be repeated every time a new sampling tool and a new configuration of the whole robotic system is investigated. Existing literature focuses on the sampling tool design [32, 33], not including the influence of the whole robotic system and the surrounding environment. On the other hand, COTS simulation tools, such as multi-body dynamics tools, allow addressing complex systems. However, using such tools for building a full design space that includes the variation of several parameters is significantly time consuming and requires a not negligible workload. Such tools would not be suited for rapid preliminary evaluation of the design space for several different configurations and boundary conditions, which is a typical need for studies that require broad trades as typically done in Concurrent Engineering Facilities. COTS simulation tools are well suited for detailed design of a very restricted number of solutions. In order to make this process more time-effective and reliable, a systematic effort is required to provide a flexible and comprehensive tool to help define the high-level requirements of a robotic system involved in sampling operations.

This paper presents MISTRAL (MultIdisciplinary deSign Tool for Robotic sAmpLing), a novel tool conceived for trade space exploration during early conceptual and preliminary design phases, where a rapid and broad evaluation is required for a very high number of configurations and boundary conditions. The tool rapidly determines the preliminary Design Envelope (DE) of a sampling apparatus to guarantee the stability condition of the whole robotic system. The tool implements a 3D (three-dimensional) analytical model capable to reproduce several scenarios, being able to accept various input parameters, such as physical and geometrical characteristics of the robotic system, properties related to the environment (i.e. gravity, physical and geometrical properties of the terrain) and features related to the sampling system (i.e. geometry, applied forces). Moreover, the model includes coupling effects among those parameters. This feature can be exploited to infer multidisciplinary high-level requirements concerning several other elements of the investigated system, such as RAs, footpads, and wheels. In this context, the DE is a graphical representation of the parameter variation, a key element for obtaining a comprehensive and rapid overview of the design space. Once the design space is defined by using MISTRAL, high-level trade-offs are conducted to narrow the range of variables. At this point, more detailed evaluation of sampling operation can be conducted on a narrower range of design variables by performing further studies that exploit more accurate models, simulation tools and experimental tests. These further activities help to identify design branches that will be the subject of a series of medium to low-level trade-offs and selections down to specific design points to analyze and test at very high detail, until a single, final solution is identified and verified.

The presented research focuses on the application of MISTRAL to landers, even if the flexibility of the tool opens the way of a future extension of its use to the analysis of rovers.

The remainder of this paper is organized according to the following structure. Section 2 presents the structure of the tool and the logic behind it, while Sect. 3 provides a detailed description of the analysis model implemented by the tool. Section 4 introduces the application of the tool to real mission data from the Phoenix Mars lander. MISTRAL has been adopted for the definition of the high-level requirements of the lander for a potential future mission to the surface of Saturn’s moon Enceladus, currently under investigation at NASA Jet Propulsion Laboratory. Section 5 presents this case study to demonstrate tool’s capabilities. Finally, Sect. 6 closes the paper with conclusions and future developments.

## Structure of the tool

MISTRAL is conceived as a tool to determine the preliminary DE of a sampling apparatus to guarantee the stability condition of the whole robotic system according to the investigated scenario.

**Fig. 1:**
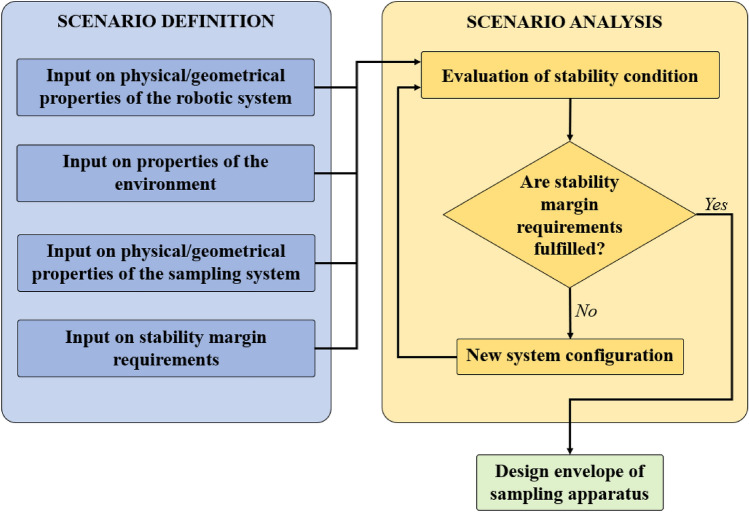
Block diagram of the tool

The process starts with the scenario definition as shown by Fig. 1, where the user inputs all relevant parameters to characterize the scenario under investigation. The first group of inputs includes physical and geometrical properties of the robotic system. In the case of considering a lander, geometrical properties include main dimensions and positioning of body, legs, and robotic arm. Physical properties include the mass of the lander and the position of its center of mass. The second group of inputs includes the properties of the environment, such as the local gravitational acceleration and the characteristics of the terrain in terms of friction and slope distribution. The third group of inputs includes the physical and geometrical properties of the sampling system. Parameters such as the geometry of the sampling system and the applied forces can be provided. The fourth group of inputs includes the required margins for the stability of the whole robotic system.

The second phase of the process is the scenario analysis. The analysis model takes the user inputs for evaluating the stability condition by solving a set of equations characterizing the equilibrium condition of the robotic system, as extensively explained in Sect. 3. In the case of considering a lander, the equilibrium condition depends on the reaction forces the terrain applies to the footpads. The reaction forces are related to several parameters, such as the lander weight which depends on its mass and on local gravitational acceleration, the morphology and the physical characteristics of the terrain (i.e. slope distribution and friction), the sampling force applied to the terrain and the way it is applied, which depends on sampling tool geometry. The tool performs an optimization process to determine the maximum allowed magnitude of the sampling force that prevents the lander changing its equilibrium state, according to the stability margins provided by the user. The stability margins are defined with respect to the reaction forces required to keep the equilibrium condition of the lander.

This process outputs the DE of the sampling apparatus according to the scenario under investigation. The DE refers to a delimited range of the parameters within which the lander keeps its static equilibrium condition with the defined stability margins.

## Analysis model

The 3D analytical model was developed to study the static equilibrium of a lander with the aim of determining the DE within which the sampling system should be designed. The model provides an indication on the maximum allowed magnitude of the sampling force that prevents the lander changing its equilibrium state. To achieve this goal, the model computes the three Cartesian components of the reaction forces acting on each lander’s footpad. The evaluation of the reaction forces enables the determination of the DE of the sampling apparatus according to the investigated scenario. The DE refers to a delimited range of the parameters within which the lander keeps its static equilibrium condition with the defined stability margins. Rotational stability is not explicitly addressed because it is usually anticipated by the lifting of the legs, which is a stability limit addressed. E.g. to get a single leg supporting most of the weight, the other legs must lift off the ground first. By preventing the legs from lifting off the ground, rotational stability issues are usually avoided. Nevertheless, rotational stability will be explicitly considered for the inclusion in the tool in future developments.

**Fig. 2:**
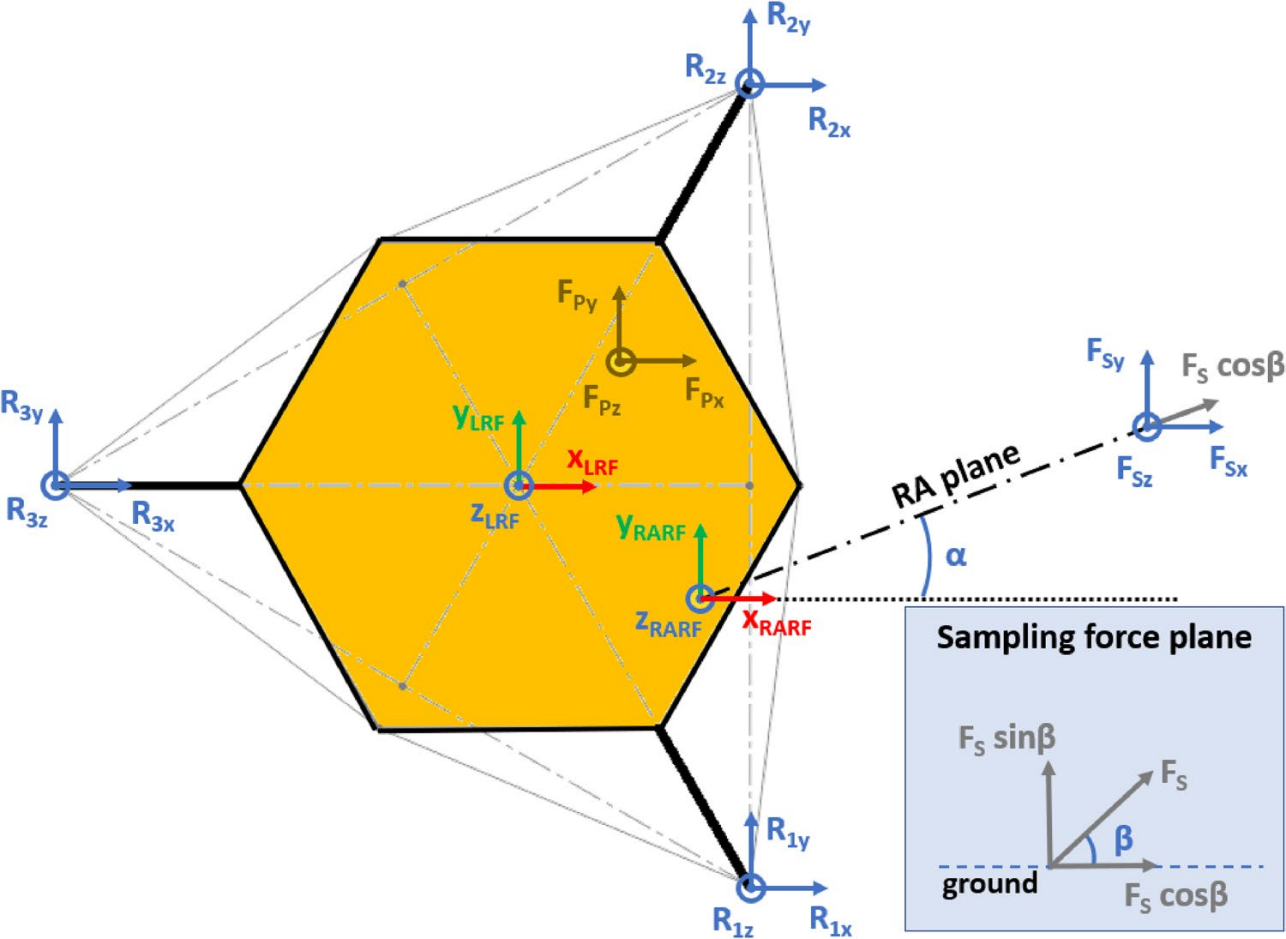
Free body diagram (*XY* plane view) for the 3-legged lander. Qualitative scheme, not to scale

**Fig. 3:**
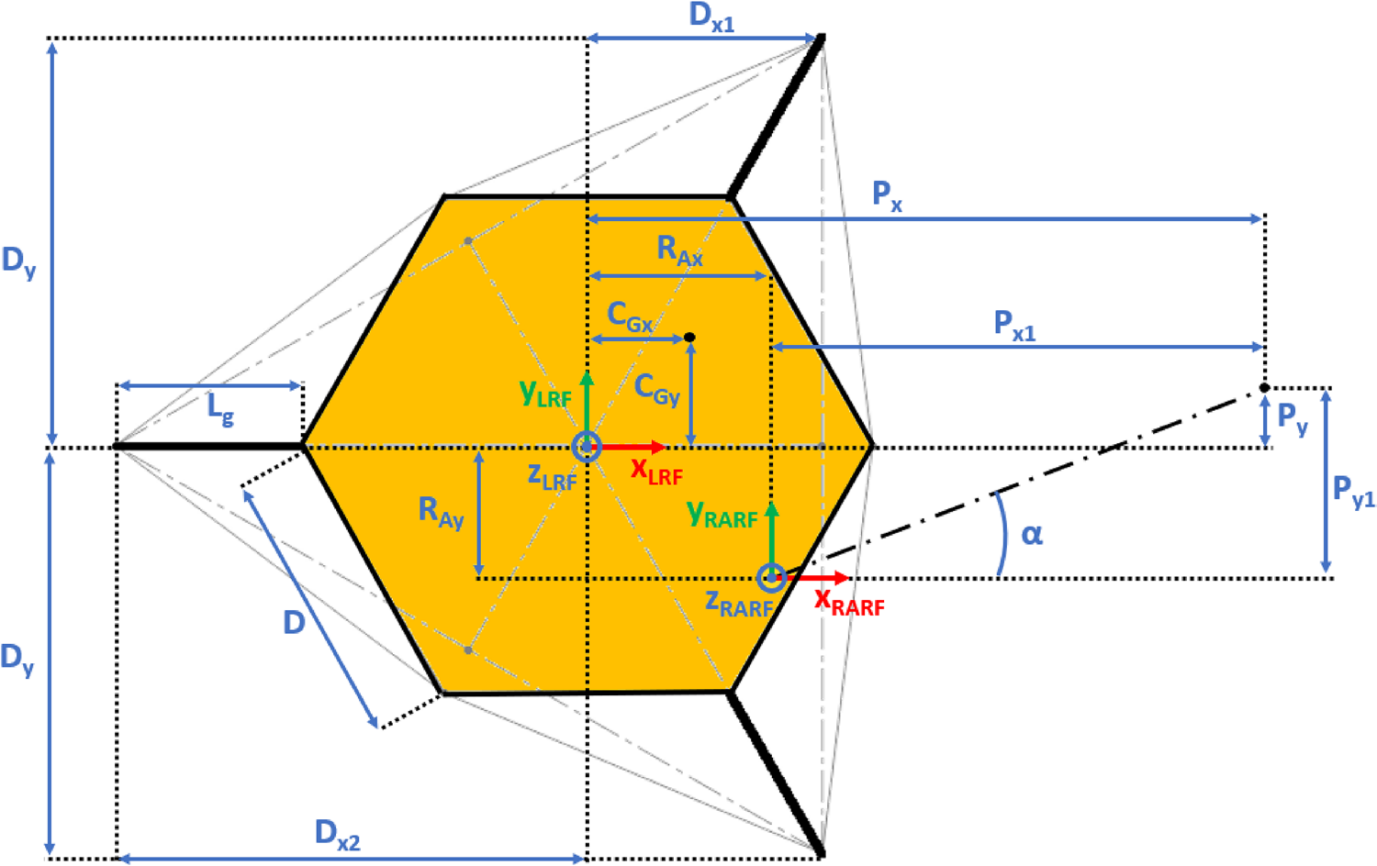
Main geometric parameters (*XY* plane view) for the 3-legged lander. Qualitative scheme, not to scale

**Fig. 4:**
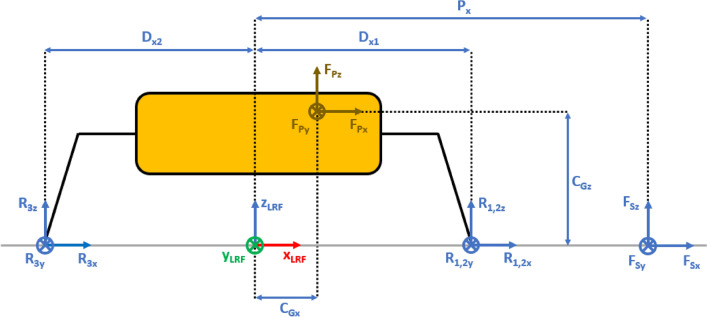
Free body diagram and main geometric parameters (*XZ* plane view) for the 3-legged lander. Qualitative scheme, not to scale

**Fig. 5:**
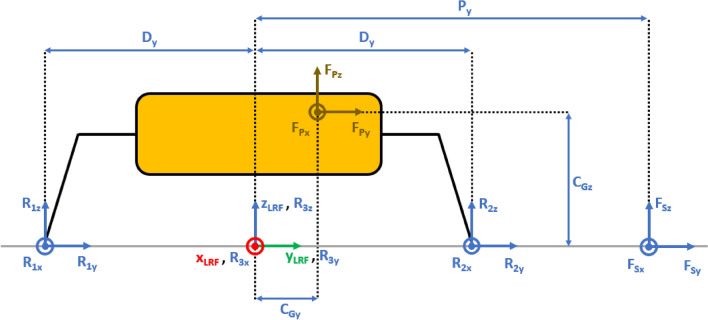
Free body diagram and main geometric parameters (*YZ* plane view) for the 3-legged lander. Qualitative scheme, not to scale

The presented approach allows the evaluation of the lander’s reaction to generic external loads having components along the three Cartesian axes, meaning that:

- The lander’s weight ${{\varvec{F}}}_{{\varvec{p}}}$ has three components. This is the effect of the ground slope angles about *X* axis $\left({\delta }_{g}\right)$ and *Y* axis $\left({\gamma }_{g}\right)$, as shown in Figs. 2, 3, 4, 5.
- The sampling force ${{\varvec{F}}}_{\mathbf{s}}$ has three components. This considers three effects. The first one is the effect of the inclination of the sampling force with respect to the ground. The second one is the effect of placing the sampling spot off-axes, as shown in Figs. 2, 3, 4, 5. The third one is the effect of the local ground slope angles about *X* axis $\left({\delta }_{s}\right)$ and *Y* axis $\left({\gamma }_{s}\right)$. The local ground slope angles are related to the local geometry of the ground at the sampling spot, which in turn influences the orientation of the sampling force.
- The Center of Gravity (${C}_{G}$) of the lander is off-axes.

As shown in Table 1, several lander configurations were historically addressed. This paper considers a 3-legged lander equipped with three footpads arranged as a regular triangle shape, and a 4-legged lander equipped with four footpads arranged as a square. This is consistent with some of the most common architectures for legged landers. To easily link the position of the footpads to the geometric characteristics of the lander body, it was assumed that the 3-legged lander has a hexagonal body, while the 4-legged lander has an octagonal body. It should be noted that any shape of the lander body can be used, since it is only an easy method to link the geometric characteristics of the lander to the position of its footpads, which in turn determines the effect of the reaction forces on the lander stability. It should be noted also that any arrangement of the legs can be studied by adjusting the position of the footpads. As an example, it would be possible to select the three main loaded legs in a legged lander with more than three legs (e.g. 4-legged lander), and switch between them to explore the DE assuming the borderline case has a single leg comes to no load (i.e. a 4-legged lander always tilts slightly, although soft regolith mediates this).

The Lander’s Reference Frame (LRF) is placed at the ground level, while the origin of LRF is aligned with the geometric center of the lander’s body. The resulting free body diagrams are shown in Figs. 2, 3, 4, 5 for the 3-legged lander and in Figs. 6, 7, 8, 9 for the 4-legged lander.

**Fig. 6:**
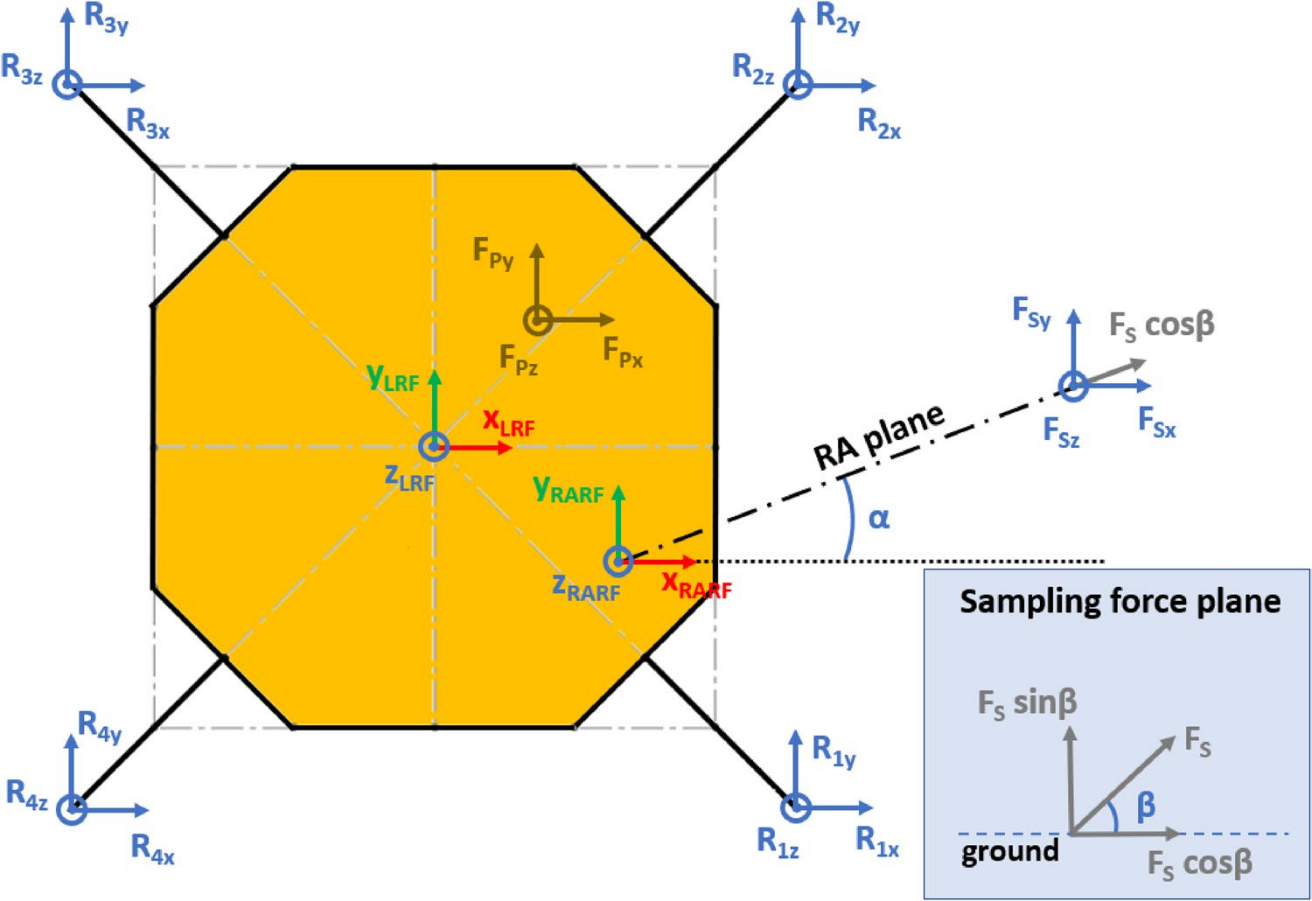
Free body diagram (*XY* plane view) for the 4-legged lander. Qualitative scheme, not to scale

**Fig. 7:**
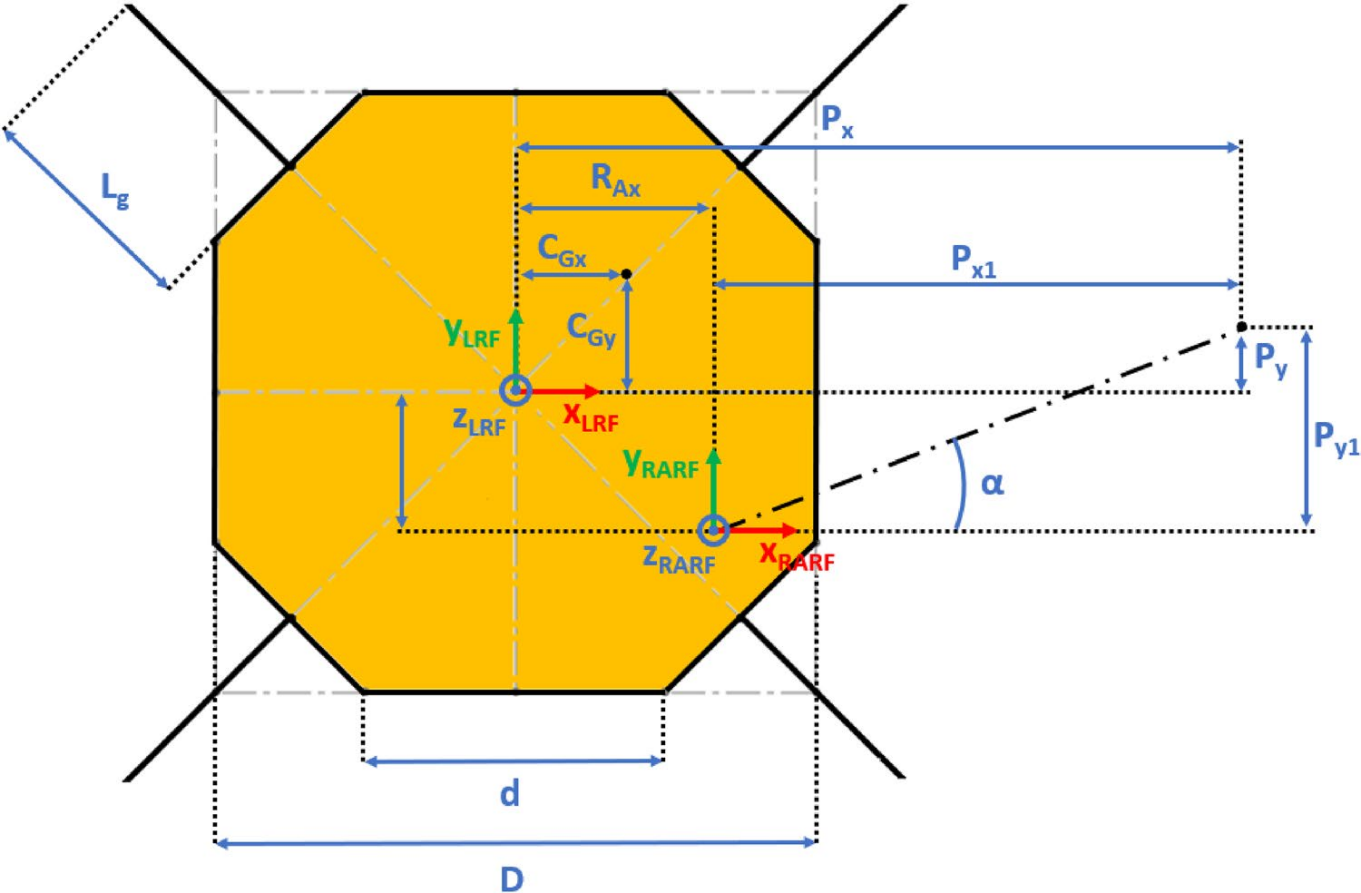
Main geometric parameters (*XY* plane view) for the 4-legged lander. Qualitative scheme, not to scale

**Fig. 8:**
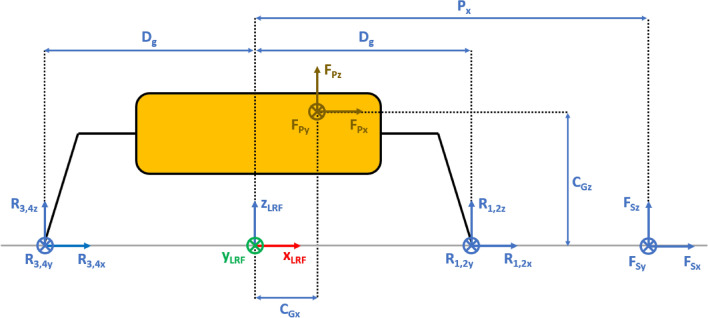
Free body diagram and main geometric parameters (*XZ* plane view) for the 4-legged lander. Qualitative scheme, not to scale

**Fig. 9:**
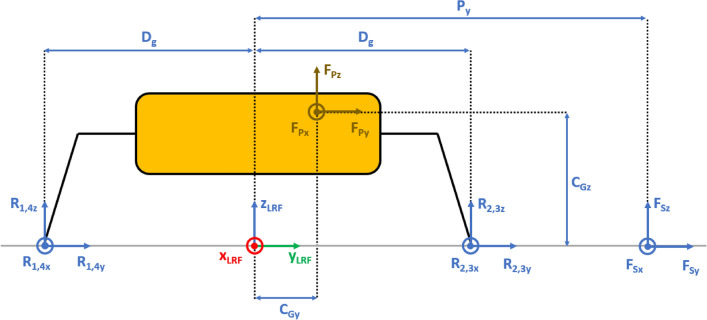
Free body diagram and main geometric parameters (*YZ* plane view) for the 4-legged lander. Qualitative scheme, not to scale

Hereafter, bold printing indicates vectors while non-bold printing indicates scalars.

${R}_{{n}_{x,y,z}}$ are the Cartesian components of the reaction force acting on the $n$-th lander’s footpad $(n=1, 2, 3, 4)$.

Given the assumption on the lander’s body shape (i.e. hexagon for the 3-legged lander, octagon for the 4-legged lander) and footpads arrangement (i.e. regular triangle for the 3-legged lander, square for the 4-legged lander), the position of the footpads is defined via the parameters ${D}_{{x}_{1,2}}$ and ${D}_{y}$ for the 3-legged lander, and ${D}_{g}$ for the 4-legged lander. It should be noted that those parameters are linked to the geometric parameters $D$, $d$ and ${L}_{g}$, defining the general configuration of the lander’s body and footpads (Fig. 2, 3, 4, 5, 6, 7, 8, 9).

${{F}_{p}}_{x,y,z}$ are the Cartesian components of the lander’s weight ${F}_{p}$. The position of the ${C}_{G}$ is defined via the geometric parameters ${{C}_{G}}_{x,y,z}$.

${{R}_{A}}_{x,y}$ define the position of the RA on the lander’s deck. Such geometric parameters define also the origin of the Robotic Arm Reference Frame (RARF).

${{F}_{s}}_{x,y,z}$ are the Cartesian components of the sampling force ${F}_{s}$. The sampling force is applied to the sampling spot, localized with respect to LRF and RARF via the geometric parameters ${P}_{x,y}$ and ${P}_{{x}_{1},{y}_{1}}$, respectively. Equation (1–2) relate geometric parameters ${P}_{x,y}$, ${P}_{{x}_{1},{y}_{1}}$ and ${{R}_{A}}_{x,y}$.

1 $${P}_{x}={{R}_{A}}_{x}+{P}_{{x}_{1}}$$

2 $${P}_{y}={{R}_{A}}_{y}+{P}_{{y}_{1}}$$

In Figs. 2 and 6, the line joining the origin of RARF to the sampling spot represents a plane containing the RA and perpendicular to the XY plane. The sampling force ${F}_{s}$ lies on the sampling force plane, which is oriented according to both the position of the RA and the local geometry of the ground at the sampling spot.

$\alpha$ is the orientation of the RA plane with respect to the *XZ* plane (i.e. a rotation about the *Z* axis of RARF), defined as $tan\alpha ={P}_{{y}_{1}}/{P}_{{x}_{1}}$.

$\beta$ is the angle of the sampling force ${F}_{s}$ with respect to the ground, located in the sampling force plane.

The vector components of the sampling force ${F}_{s}$ are computed by defining the local ground slope angles about *X* axis $\left({\delta }_{s}\right)$ and *Y* axis $\left({\gamma }_{s}\right)$ of RARF. A rotation matrix is defined for the rotation about the X axis, Y axis and the Z axis, according to Eq. (3–5) respectively. The rotation matrices are applied to the base sampling force vector ${\varvec{S}}={F}_{s} \left[cos\beta , 0,sin\beta \right]$ to obtain its components, according to Eq. (6).

3 $${\Gamma }_{s}=\left[\begin{array}{ccc}1& 0& 0\\ 0& cos{\delta }_{s}& -sin{\delta }_{s}\\ 0& sin{\delta }_{s}& cos{\delta }_{s}\end{array}\right]$$

4 $${\Delta }_{s}=\left[\begin{array}{ccc}{cos\gamma }_{s}& 0& sin{\gamma }_{s}\\ 0& 1& 0\\ -sin{\gamma }_{s}& 0& {cos\gamma }_{s}\end{array}\right]$$

5 $${\rm A}_{s}=\left[\begin{array}{ccc}cos\alpha & -sin\alpha & 0\\ sin\alpha & cos\alpha & 0\\ 0& 0& 1\end{array}\right]$$

6 $${{\varvec{F}}}_{{\varvec{s}}}={\rm A}_{s} {\Gamma }_{s} {\Delta }_{s} {\varvec{S}}$$

The vector components of the lander’s weight ${F}_{p}$ are computed by defining the general ground slope angles about *X* axis $\left({\delta }_{g}\right)$ and *Y* axis $\left({\gamma }_{g}\right)$ of LRF. A rotation matrix is defined for both the rotation about the *X* axis and the *Y* axis, according to Eqs. (7–8), respectively. The rotation matrices are applied to the base weight vector ${\varvec{W}}=\left[0, 0, -{F}_{w}\right]$ to obtain the vector components of the lander’s weight, according to Eq. (9). ${F}_{w}=mg$ is the base weight force, where $m$ is the lander’s mass and $g$ is the gravitational acceleration.

7 $${\Gamma }_{g}=\left[\begin{array}{ccc}1& 0& 0\\ 0& cos{\delta }_{g}& -sin{\delta }_{g}\\ 0& sin{\delta }_{g}& cos{\delta }_{g}\end{array}\right]$$

8 $${\Delta }_{g}=\left[\begin{array}{ccc}{cos\gamma }_{g}& 0& sin{\gamma }_{g}\\ 0& 1& 0\\ -sin{\gamma }_{g}& 0& {cos\gamma }_{g}\end{array}\right]$$

9 $${{\varvec{F}}}_{{\varvec{p}}}={\Gamma }_{g} {\Delta }_{g} {\varvec{W}}$$

Equations (10–11) show the vector form of the systems of equations to compute the reaction forces acting on the footpads for a 3-legged and a 4-legged lander, respectively. Lines 1–2 in Eqs. (10–11) derive from the free body diagrams and represent equilibrium conditions with respect to LRF.

**Fig. 10:**
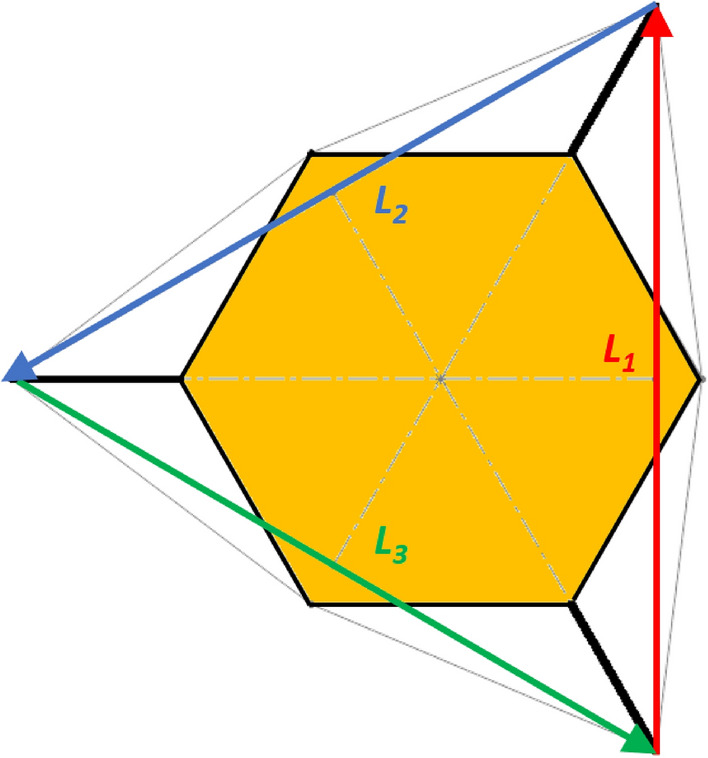
Vectors joining the lander’s footpads for the 3-legged lander. Qualitative scheme, not to scale

Line 3 in Eq. (10) represents the geometric conditions imposed by assuming that the lander behaves as a rigid body. This means that the relative distance between the footpads does not change. The geometric conditions bring to the assumption that the regular triangle shape does not change. There are several ways to define a regular triangle. The one selected is to impose that each side of the triangle has a constant length equal to the other ones, as shown in Fig. 10.

**Fig. 11:**
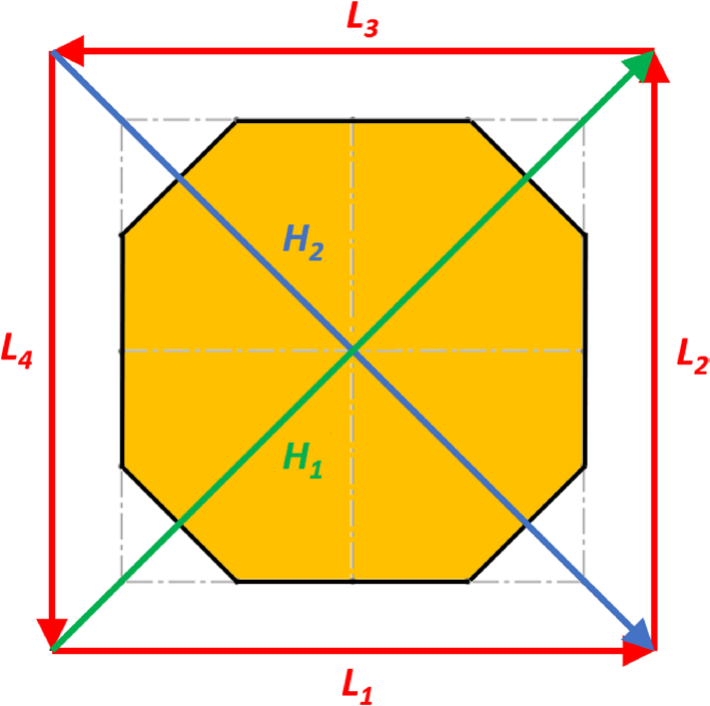
Vectors joining the lander’s footpads for the 4-legged lander. Qualitative scheme, not to scale

Lines 3–5 in Eq. (11) represent the geometric conditions imposed by assuming that the lander behaves as a rigid body. This means that the relative distance between the footpads does not change. The geometric conditions bring to the assumption that the square shape does not change. There are several ways to define a square. The one selected is to impose that each diagonal has a constant length equal to the other one (line 3). Moreover, the two diagonals are imposed to be perpendicular each other, similarly for the sides of the square (line 4), as shown in Fig. 11. Finally, all the footpads are imposed to lie on the same plane (line 5). This is obtained by imposing that the determinant of matrix $A$ (i.e. the matrix defining the equation of a plane passing though the four footpads) is equal to zero.

10 $$\left\{\begin{array}{c}\begin{array}{c}\sum_{n=1}^{3}{{\varvec{R}}}_{{\varvec{n}}}+{{\varvec{F}}}_{{\varvec{s}}}+{{\varvec{F}}}_{{\varvec{p}}}=0\\ \sum_{n=1}^{3}{{{\varvec{D}}}_{{\varvec{n}}}\times {\varvec{R}}}_{{\varvec{n}}}+{{\varvec{D}}}_{{\varvec{s}}}\times {{\varvec{F}}}_{{\varvec{s}}}+{{\varvec{D}}}_{{\varvec{p}}}\times {{\varvec{F}}}_{{\varvec{p}}}=0\\ {\Vert {{\varvec{L}}}_{1}\Vert }_{2}={\Vert {{\varvec{L}}}_{2}\Vert }_{2}={\Vert {{\varvec{L}}}_{3}\Vert }_{2}=c\end{array}\end{array}\right.$$

$${{\varvec{R}}}_{{\varvec{n}}}=\left({R}_{{n}_{x}},{R}_{{n}_{y}},{R}_{{n}_{z}}\right) $$
Reaction force vector acting on n-th footpad
$${{\varvec{F}}}_{{\varvec{s}}}=\left({F}_{{s}_{x}},{F}_{{s}_{y}},{F}_{{s}_{z}}\right)$$
Sampling force vector
$${{\varvec{F}}}_{{\varvec{p}}}=\left({F}_{{p}_{x}},{F}_{{p}_{y}},{F}_{{p}_{z}}\right)$$
Lander weight vector
$${{\varvec{D}}}_{1}={{\varvec{D}}}_{2}=\left({D}_{{x}_{1}},{D}_{y},0\right)$$
Footpads position vector 1
$${{\varvec{D}}}_{3}=\left({D}_{{x}_{2}},0, 0\right)$$
Footpads position vector 2
$${{\varvec{D}}}_{{\varvec{s}}}=\left({P}_{x},{P}_{y},0\right)$$
Sampling spot position vector
$${{\varvec{D}}}_{{\varvec{p}}}=\left({{C}_{G}}_{x},{{C}_{G}}_{y},{{C}_{G}}_{z}\right)$$
Center of Gravity position vector
$${D}_{{x}_{1}}=\left(D+{L}_{g}\right)sin\frac{\pi }{6}  $$
*X* Coordinate of footpads 1–2, dependent on lander geometric parameters $$D,{L}_{g} $$(Figs. 2, 3, 4, 5)
$${D}_{{x}_{2}}=\left(D+{L}_{g}\right)  $$
*X* Coordinate of footpad 3, dependent on lander geometric parameters $$D,{L}_{g} $$ (Figs. 2, 3, 4, 5)
$${D}_{y}=\left(D+{L}_{g}\right)sin\frac{\pi }{3} $$
*Y* Coordinate of footpads 1–2, dependent on lander geometric parameters $D,{L}_{g}$ (Figs. 2, 3, 4, 5)
$$c=2{D}_{y}$$
Length of each side of the regular triangle having the footpads as vertices

11 $$\left\{\begin{array}{c}\begin{array}{c}\sum_{n=1}^{4}{{\varvec{R}}}_{{\varvec{n}}}+{{\varvec{F}}}_{{\varvec{s}}}+{{\varvec{F}}}_{{\varvec{p}}}=0\\ \sum_{n=1}^{4}{{{\varvec{D}}}_{{\varvec{n}}}\times {\varvec{R}}}_{{\varvec{n}}}+{{\varvec{D}}}_{{\varvec{s}}}\times {{\varvec{F}}}_{{\varvec{s}}}+{{\varvec{D}}}_{{\varvec{p}}}\times {{\varvec{F}}}_{{\varvec{p}}}=0\\ {\Vert {{\varvec{H}}}_{1}\Vert }_{2}={\Vert {{\varvec{H}}}_{2}\Vert }_{2}=b\\ {{\varvec{H}}}_{1}\bullet {{\varvec{H}}}_{2}={{\varvec{L}}}_{1}\bullet {{\varvec{L}}}_{2}={{\varvec{L}}}_{3}\bullet {{\varvec{L}}}_{4}=0\\ \left|\begin{array}{c}A\end{array}\right|=0\end{array}\end{array}\right.$$

$${{\varvec{R}}}_{{\varvec{n}}}=\left({R}_{{n}_{x}},{R}_{{n}_{y}},{R}_{{n}_{z}}\right) $$
Reaction force vector acting on n-th footpad
$${{\varvec{F}}}_{{\varvec{s}}}=\left({F}_{{s}_{x}},{F}_{{s}_{y}},{F}_{{s}_{z}}\right)$$
Sampling force vector
$${{\varvec{F}}}_{{\varvec{p}}}=\left({F}_{{p}_{x}},{F}_{{p}_{y}},{F}_{{p}_{z}}\right)$$
Lander weight vector
$${{\varvec{D}}}_{1}={{\varvec{D}}}_{2}={{\varvec{D}}}_{3}={{\varvec{D}}}_{4}=\left({D}_{g},{D}_{g}, 0\right)$$
Footpads position vector
$${{\varvec{D}}}_{{\varvec{s}}}=\left({P}_{x},{P}_{y},0\right)$$
Sampling spot position vector
$${{\varvec{D}}}_{{\varvec{p}}}=\left({{C}_{G}}_{x},{{C}_{G}}_{y},{{C}_{G}}_{z}\right)$$
Center of Gravity position vector
$${D}_{g}=\left(c+{L}_{g}\right)sin\frac{\pi }{4}  $$
*X, Y* coordinate of footpads 1–4 dependent on lander geometric parameters$D,\,d,{L}_{g}$ (Figs. 6, 7, 8, 9)
$$b=2\left(c+{L}_{g}\right) $$
Length of the diagonal of the square having the footpads as vertices, dependent on lander geometric parameters $D,\,d,{L}_{g}$ (Figs. 6, 7, 8, 9)
$$c=\frac{D}{2}\left(\sqrt{2}-cos\frac{\pi }{4}\right)+\frac{d}{2}cos\frac{\pi }{4}$$
Constant adopted to simplif the notation, dependent on lander geometric parameters $D,\,d$ (Figs. 6, 7, 8, 9)

In order to exploit the geometric conditions in Eqs. (10–11), it is required to link those equations with the variables to be computed (i.e. the reaction forces $R$). For this reason, the point of contact between each footpad and the ground was modeled via three springs along the three Cartesian axes. It is assumed that the springs have all the same constant stiffness $k$. Given this assumption, it was found that the physical solution of Eqs. (10–11) is independent on the value of the spring stiffness. The reactions forces can be related to the displacement $d$ of the lander’s footpads via Eq. (12).

12 $$R=k d$$

The goal is the evaluation of the maximum allowed magnitude of the sampling force ${F}_{s}$ that prevents the lander changing its equilibrium state. Therefore, the sampling force ${F}_{s}$ represents the independent variable, while the reaction forces represent the dependent variable. Equations (10–11) were symbolically solved to get the explicit dependence of the reaction forces from the sampling force, ${R}_{n}=f({F}_{s})$. The maximum allowed sampling force is defined such that the reaction forces ${R}_{n}$ do not overcome a certain pre-defined limit force. According to Eqs. (13–14), the limit force is computed by defining a margin for the reaction forces. The limit force ${L}_{xy}$ is applied to prevent the lander from sliding in the *XY* plane. The limit force ${L}_{z}$ is applied to prevent the lander from lifting off the *XY* plane (i.e. the ground).

The limit force ${L}_{xy}$ is defined by applying a margin ${M}_{xy}$ with respect to the friction force ${F}_{a}$, which is the boundary for the incipient motion of the lander. According to Eq. (15), the friction force is defined with respect to the *Z* component of the $n$-th reaction force through the coefficient of friction $\mu$ between the lander’s footpad and the ground. The limit force ${L}_{z}$ is defined by applying a margin ${M}_{z}$ with respect to a pre-defined minimum value for the *Z* component of the reaction force $({K}_{z})$.

Margins ${M}_{xy}$ and ${M}_{z}$ can get any positive real value, where a value equal to 0 means a margin of 0%, a value of 1 means a margin of 100%, etc.

13 $${L}_{xy}=\frac{{F}_{a}}{\left(1+{M}_{xy}\right)}$$

14 $${L}_{z}={K}_{z}\left(1+{M}_{z}\right)$$

15 $${F}_{a}=\mu {R}_{{n}_{z}}$$

The limit force ${L}_{xy}$ depends on the sampling force ${F}_{s}$ through the *Z* component of the reaction force ${R}_{{n}_{z}}$. On the other hand, the limit force ${L}_{z}$ is pre-defined.

The maximum allowed magnitude of the sampling force ${F}_{s}$, named ${F}_{{s}_{max}}$, is evaluated by minimizing the objective functions defined by Eqs. (16–17).

16 $${J}_{xy}={\left({L}_{xy}-{R}_{{n}_{xy}}\right)}^{2}$$

17 $${J}_{z}={\left({L}_{z}-{R}_{{n}_{z}}\right)}^{2}$$

where $${R}_{{n}_{xy}}=\sqrt{{R}_{{n}_{x}}^{2}+{R}_{{n}_{y}}^{2}}$$

**Fig. 12:**
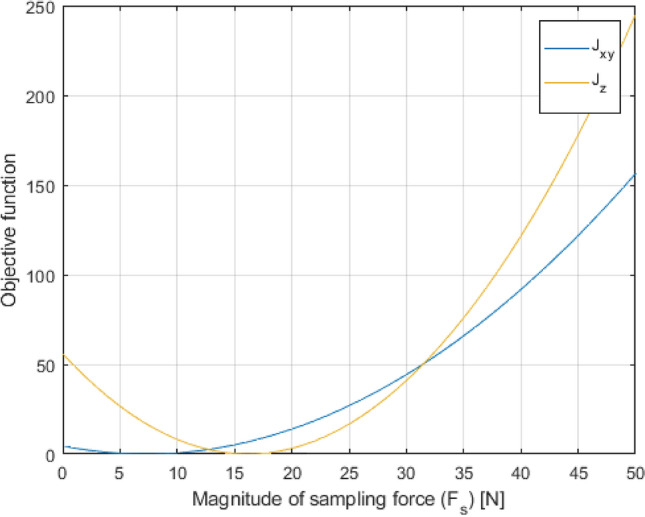
Example of convex objective functions

The objective functions are convex, meaning that they have a single global minimum (Fig. 12). The minimization problem is defined according to Eqs. (18–19).

18 $${\left({F}_{{s}_{max}}\right)}_{xy}=\underset{{F}_{s}}{min}{J}_{xy}\left({F}_{s}\right)\,such \,that\,{F}_{{s}_{1}}<{F}_{s}<{F}_{{s}_{2}}$$

19 $${\left({F}_{{s}_{max}}\right)}_{z}=\underset{{F}_{s}}{min}{J}_{z}\left({F}_{s}\right)\,such\, that\,{F}_{{s}_{1}}<{F}_{s}<{F}_{{s}_{2}}$$

The minimization problem is solved by using the MATLAB function *fminbnd*, which finds the minimum of a single-variable function on a fixed interval [34, 35]. As a conservative approach, the minimum value among the two computed by solving the minimization problems is selected, according to Eq. (20).

20 $${F}_{{s}_{max}}=min\left({\left({F}_{{s}_{max}}\right)}_{xy} , {\left({F}_{{s}_{max}}\right)}_{z}\right)$$

${F}_{{s}_{max}}$ is obtained with respect to a specific condition, since all the parameters (i.e. environmental, physical, geometrical) except the sampling force ${F}_{s}$ are defined prior solving the minimization problem. At different environmental, physical or geometrical conditions correspond different values of ${F}_{{s}_{max}}$. By exploring the parameters’ space, it is possible to derive the DE of the lander. Three kinds of parameters are used to define the DE: dependent, independent and boundary.

The dependent parameters represent the output whose variation is used to determine the static equilibrium condition. In this case, the dependent parameters are the reaction forces ${R}_{n}$ acting on the lander’s footpads. The margin ${M}_{xy}$ on the value of the friction force ${F}_{a}$ is used to define the limit ${L}_{xy}$ of the DE in the XY plane. In fact, the friction force determines the boundary for the incipient motion of the lander. On the other hand, the margin ${M}_{z}$ on the null value of the *Z* component of the reaction force is used to define the limit ${L}_{z}$ of the DE along the *Z* axis. In fact, the null value represents the boundary for the incipient lifting of the lander.

The independent parameters represent inputs that affect the dependent parameters, in this case represented by the magnitude of the sampling force ${F}_{s}$.

The boundary parameters represent all the inputs not directly involved in the minimization process. Such parameters include.

- Environmental parameters
  - Gravitational acceleration $$\left(g\right)$$
  - Slope of the ground about *X*, *Y* axes $\left({\delta }_{g},{\gamma }_{g}\right)$
  - Local slope of the ground about *X*, *Y* axes at the sampling spot $\left({\delta }_{s},{\gamma }_{s}\right)$
  - Footpad-to-ground coefficient of friction $\left(\mu \right)$
- Physical and geometrical parameters of the lander
  - Mass $$\left(m\right)$$
  - Length of the leg’s projection in the *XY* plane $\left({L}_{g}\right)$
  - Length of the body’s side $\left(D\right)$
  - Cartesian components of the ${C}_{G}$ position $\left({C}_{{G}_{x,y,z}}\right)$
  - Margin on the reaction forces $\left({M}_{xy,z}\right)$
- Physical and geometrical parameters of the sampling
  - Cartesian components of the position of the sampling spot $\left({P}_{x,y}\right)$
  - Inclination of the sampling force with respect to the ground $\left(\beta \right)$

**Fig. 13:**
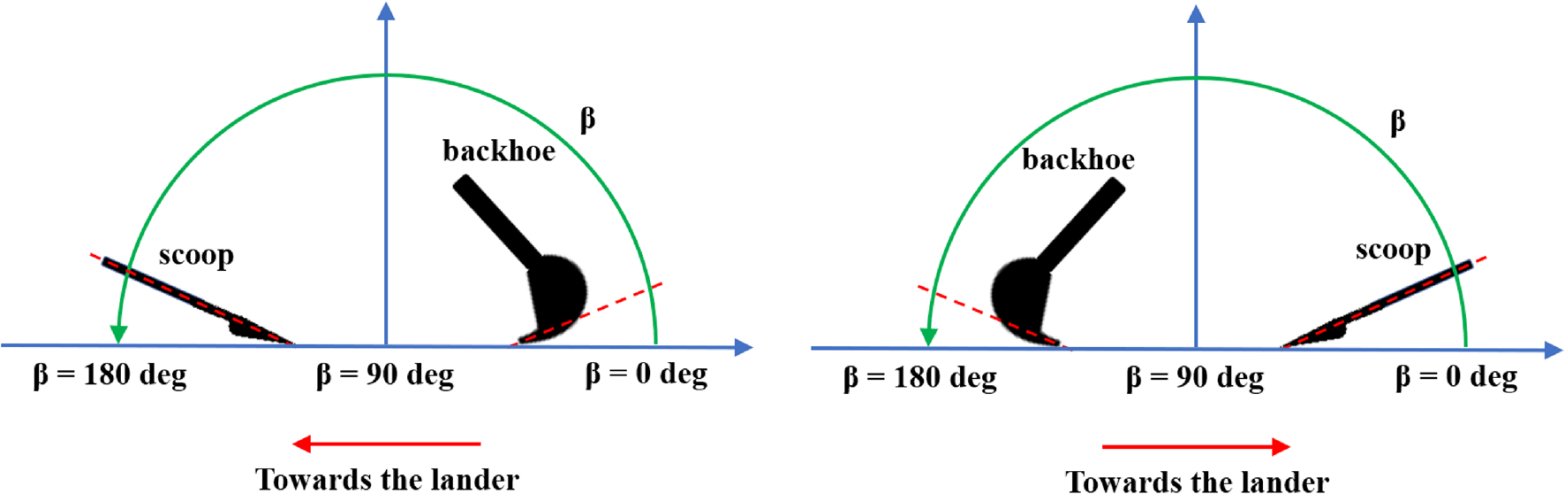
Some sampling system configurations that might be explored by changing the $\beta$ angle

By changing the boundary parameters, it is possible to explore several environmental conditions as well as several physical and geometrical configurations related to both lander and sampling (Fig. 13). Among potential sampling tools, it should be mentioned that highly dynamic systems such as drills are probably less suited to the application of the technique presented in this paper.

## Application to NASA’s Phoenix Mars lander mission

This Section presents the application of the tool to real mission data from the NASA’s Phoenix Mars lander.

**Fig. 14:**
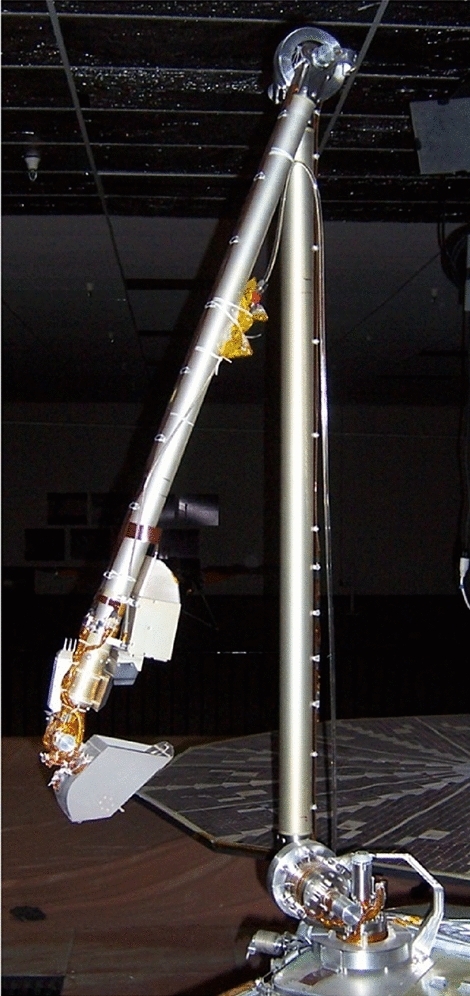
Engineering model of the Phoenix lander RA and ISAD. Credits: NASA/University of Arizona

The Phoenix lander touched down on 25 May 2008 in the Green Valley, a high latitude Mars region, and operated until 2 November 2008, acquiring data during 152 sols (i.e. Mars days) of operations. The Phoenix lander was equipped with a 2.4 m RA with an Icy Soil Acquisition Device (ISAD) (Fig. 14). The ISAD is composed by a scoop capable of excavating trenches, a scraper blade for hard soils and a rasp tool [36, 37].

Several trenches were excavated and resistive forces during backhoe operations were derived. The highest forces registered during mission operations are shown in Table 2.

**Table 2:** Force peaks registered during Phoenix Mars lander operations

Name	Force magnitude [N]	Force component	References
Dodo-Goldilocks 116–1	70	Normal to surface	[36, 37]
Dodo-Goldilocks 116–2	75	Normal to surface	[38]
Stone Soup 74, 76, 85, 88	100	Total force in excavation plane	[38]

The name highlights the denomination of the excavation site followed by a number indicating the reference sol (i.e. Mars day)

The procedure aims to check that the force peaks lie inside the DE of the Phoenix lander. Therefore, the first step is the computation of the DE. The values of the model parameters are shown in Table 3.

**Table 3:** Values of the model parameters for the Phoenix lander

Constant parameters	
**Environmental parameters**	**Value**
Gravitational acceleration $\left(g\right)$	3.72 m/s^2^
Slope of the ground about *X* axis $\left({\delta }_{g}\right)$	0°
Local slope of the ground about *X* axis at the sampling spot $\left({\delta }_{s}\right)$	0°
Local slope of the ground about *Y* axis at the sampling spot $\left({\gamma }_{s}\right)$	0°
**Physical and geometrical parameters of the lander**	
Length of the leg’s projection in the *XY* plane $\left({L}_{g}\right)$	0.2 m
Length of the body’s side $\left(D\right)$	0.75 m
[*X*, *Y*, *Z*] components of the ${C}_{G}$ position $\left({C}_{{G}_{x,y,z}}\right)$	(0, 0, 1) m
Margin on the reaction forces $\left({M}_{xy,z}\right)$	(1, 1)
Mass $\left(m\right)$	350 kg
**Physical and geometrical parameters of the sampling**	
Inclination of the sampling force with respect to the ground $\left(\beta \right)$	33.9°

Variable parameters	
**Environmental parameters**	**Range**
Slope of the ground about *Y* axis $\left({\gamma }_{g}\right)$	(0 ÷ 20)°
Footpad-to-ground coefficient of friction $\left(\mu \right)$	0.65 ÷ 0.93
**Physical and geometrical parameters of the sampling**	
[*X*, *Y*] components of the position of the sampling spot $\left({P}_{{x}_{1},{y}_{1}}\right)$	Derived according to the definition of the RA workspace

It was assumed that the body of the Phoenix lander has a regular hexagonal shape. Given this assumption, the length of the body’s side $\left(D\right)$ was derived from [39].

The length of the leg’s projection in the *XY* plane $\left({L}_{g}\right)$ was assumed from [37] by considering a proportion with respect to the lander’s deck diameter [39].

The *X*, *Y* components of the ${C}_{G}$ position $\left({C}_{{G}_{x,y}}\right)$ were assumed coincident with the LRF origin, while the Z component $\left({C}_{{G}_{z}}\right)$ was assumed by considering the lander’s height [39].

The lander’s mass was derived from [39].

The sampling system of the Phoenix lander is a backhoe. The inclination of the sampling force with respect to the ground $\left(\beta \right)$ was derived from [38] and converted according to the convention of Fig. 13.

A 100% margin on *XY, Z *components of the reaction forces $\left({M}_{xy,z}\right)$ were assumed.

The slope of the ground was assumed only about *Y* axis $\left({\gamma }_{g}\right)$, while the slope about X axis $\left({\delta }_{g}\right)$ was assumed negligible. The local slopes at the sampling spot (${\gamma }_{s}$, ${\delta }_{s}$) were also assumed negligible.

The footpad-to-ground coefficient of friction $\left(\mu \right)$ was derived from the angle of internal friction of the soil $\left(\varphi \right)$ through the Coulomb’s law $\mu =tan\varphi$ [40]. Since the angle of internal friction is 38°  ± 5° [38], the coefficient of friction was found varying in the range 0.65 ÷ 0.93.

The sampling spot was assumed varying within the RA workspace derived from [37]. The workspace is delimited by an upper and a lower ends, and by an inner and an outer circular sectors centered in the RARF origin (Fig. 16). The inner and outer circular sectors have a radius of 1.52 m and 2.14 m, respectively. The radius of the outer circular sector was derived from [37], while the radius of the inner circular sector was assumed by considering a proportion with respect to the outer radius. The upper and lower ends were assumed spanning across a 90° angle.

**Fig. 15:**
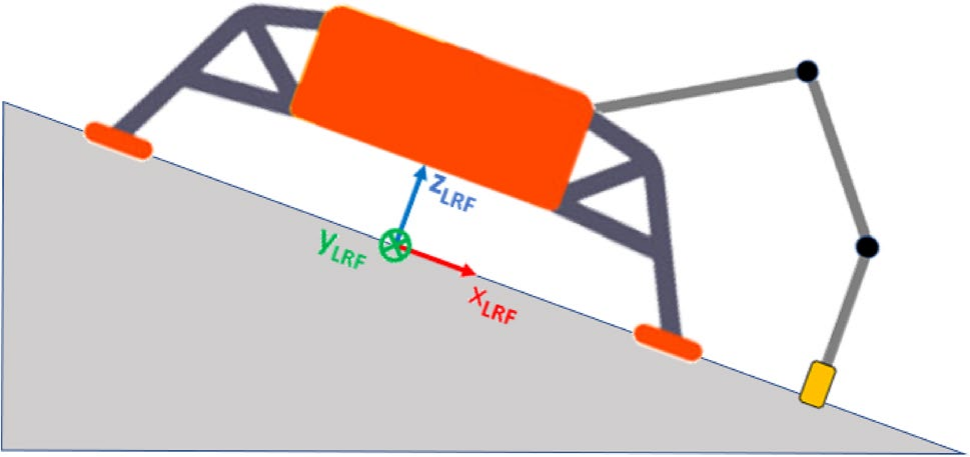
Phoenix lander worst-case configuration. The lander is inclined about the *Y* axis and pulled downhill during backhoe operations. Qualitative scheme, not to scale

**Fig. 16:**
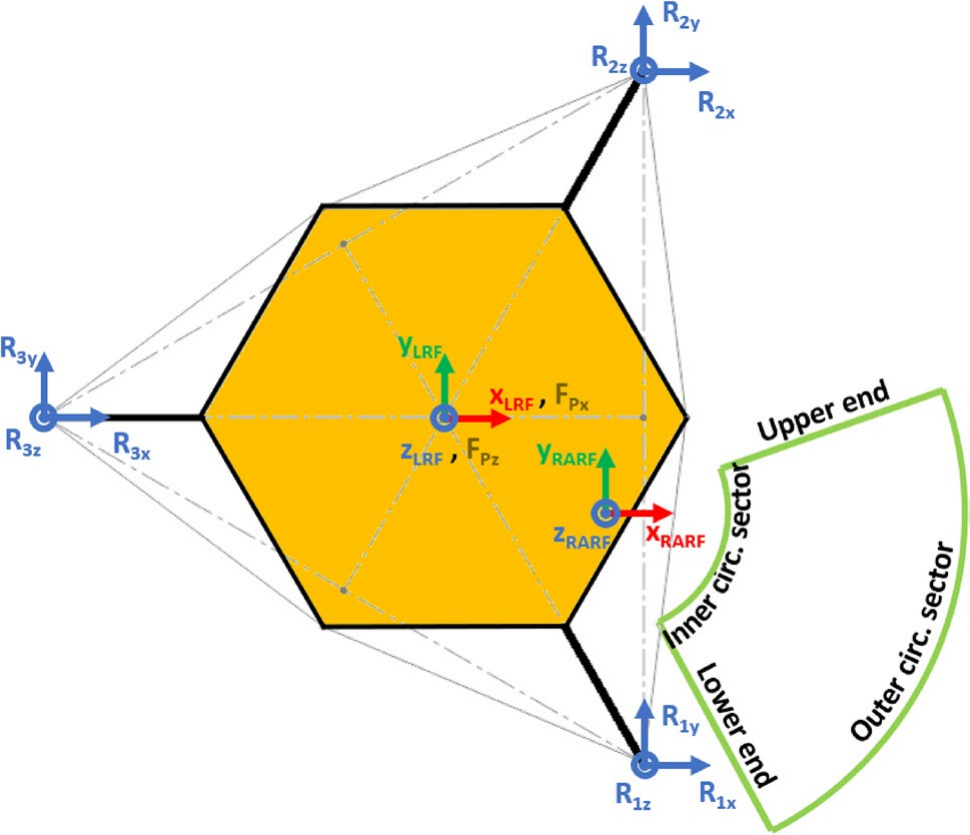
Phoenix lander configuration. The sampling spot lies in the green area. Qualitative scheme, not to scale

The configuration assumed for the Phoenix lander is shown in Figs. 15, 16. Such a configuration might be considered a worst-case scenario, since the lander is pulled downhill by the RA.

**Fig. 17:**
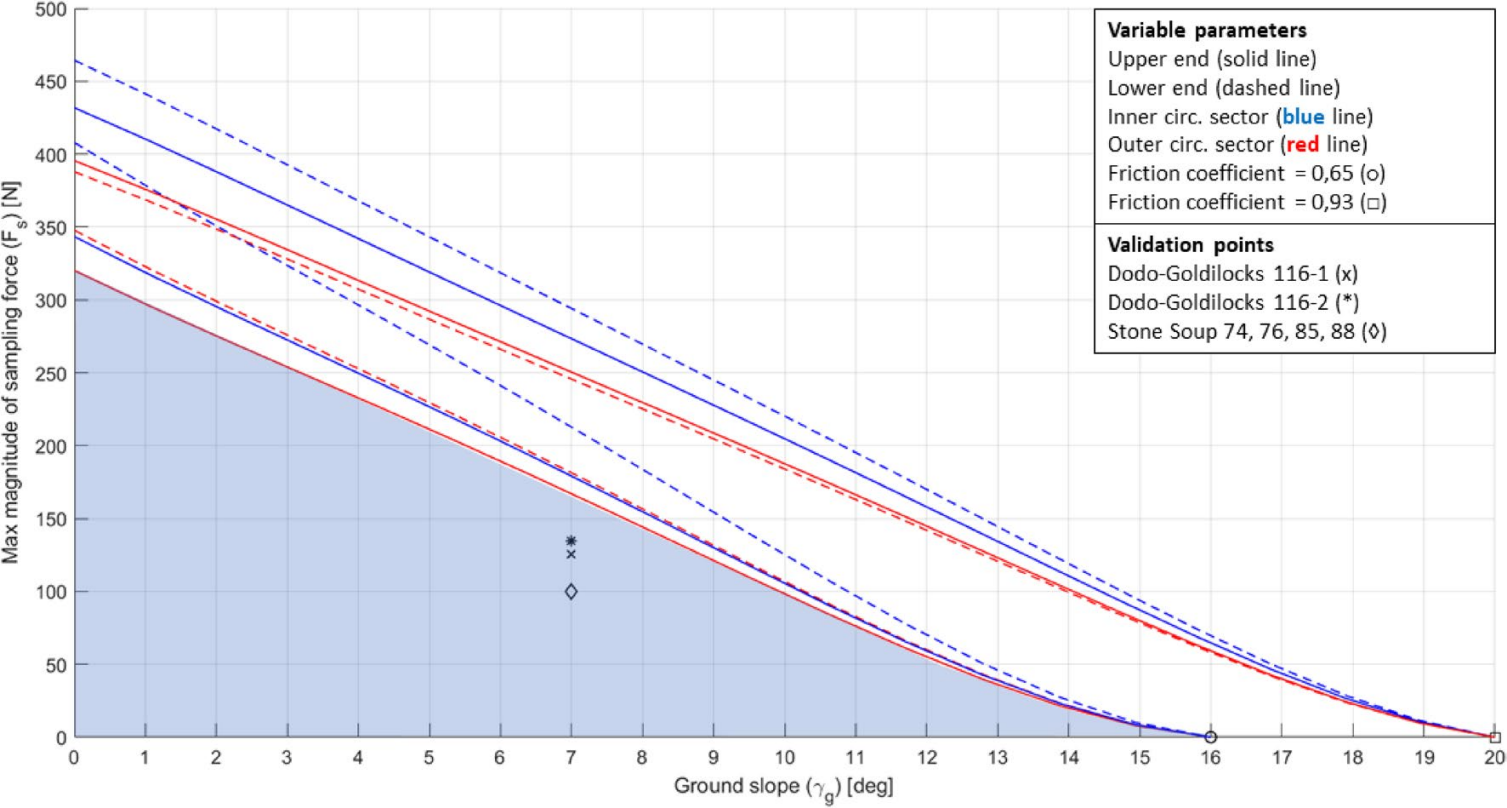
DE of the Phoenix lander together with the mission data points

The obtained DE is shown in Fig. 17, where each line represents the locus of the points where the magnitude of the sampling force ${F}_{s}$ is maximum, according to the defined margin. The DE was defined with respect to the ground slope since this environmental parameter is particularly crucial for surface operations. Several max-${F}_{s}$ lines were obtained by changing the variable parameters within the defined ranges. The DE is defined as the area underlying a max-${F}_{s}$ line. The DE of the Phoenix lander was selected as the area underlying the lowest max-${F}_{s}$ line, considered as a worst-case scenario (light blue area in Fig. 17).

In order to plot the mission data points reported in Table 2, they have to be first converted to represent the total magnitude of the sampling force, according to the input required by the DE. Since the original Stone Soup point represents the total force in the excavation plane, it was assumed that it already represents the required input. On the other hand, the original Dodo–Goldilocks points ${F}_{{s}_{o}}$ represent the normal force, so they have to be converted into the required input through the inclination of the sampling force with respect to the ground $\left(\beta \right)$. The new value ${F}_{{s}_{n}}$ is obtained through the equation ${F}_{{s}_{n}}={F}_{{s}_{o}}/sin\beta$. The values of the plotted data points are reported in Table 4.

**Table 4:** Mission data point values converted according to the required input for the DE

Name	Force magnitude [N]
Dodo–Goldilocks 116–1	125.5
Dodo–Goldilocks 116–2	134.5
Stone soup 74, 76, 85, 88	100

The ground slope selected to plot the validation points has a value of 7°, reported as the average value at the landing site of the Phoenix lander [41, 42].

Figure 17 shows that the mission data points lie inside the DE of the Phoenix lander, confirming the quality of the approach, even considering the worst-case scenarios.

## Application to a potential future mission

This Section presents the application of the tool to a potential future landing mission to the surface of Saturn’s moon Enceladus with the aim to collect surface samples by using a scoop-like sampling system. Such a potential future mission is currently under investigation at NASA Jet Propulsion Laboratory. In such a context, the tool was adopted to derive high-level requirements on the sampling system and to infer high-level requirements on the lander system [28].

Enceladus is one of the most promising places in the Solar System that might potentially host life beyond Earth. The Cassini mission strongly suggested the presence of hydrothermal activity and observed material from the subsurface ocean being ejected by plumes and then settling on the surface [28].

The very low surface gravity of Enceladus represents a new challenge for surface sampling. Even small forces applied to the lander could weaken its equilibrium state, potentially causing the lander to lift or slide downhill. Therefore, a critical task is the evaluation of the effect of the forces the sampling system might apply to the lander while performing the sampling operations.

Two lander configurations were studied, a 3-legged and a 4-legged lander. A sensitivity analysis was conducted to determine which among the boundary parameters have low sensitivity and can thus be considered constant. The boundary parameters having a high sensitivity were then considered variable parameters. By exploring the space of the variable parameters, it is possible to derive the DE.

**Fig. 18:**
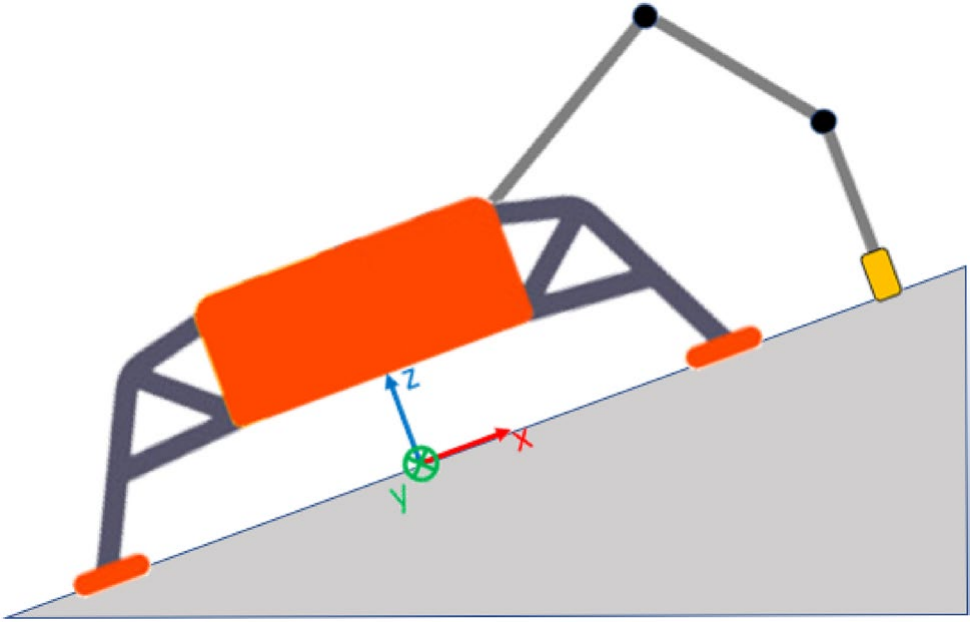
Enceladus lander worst-case configuration. The lander is inclined about the *Y* axis and pushed downhill during scooping operations. Qualitative scheme, not to scale

**Fig. 19:**
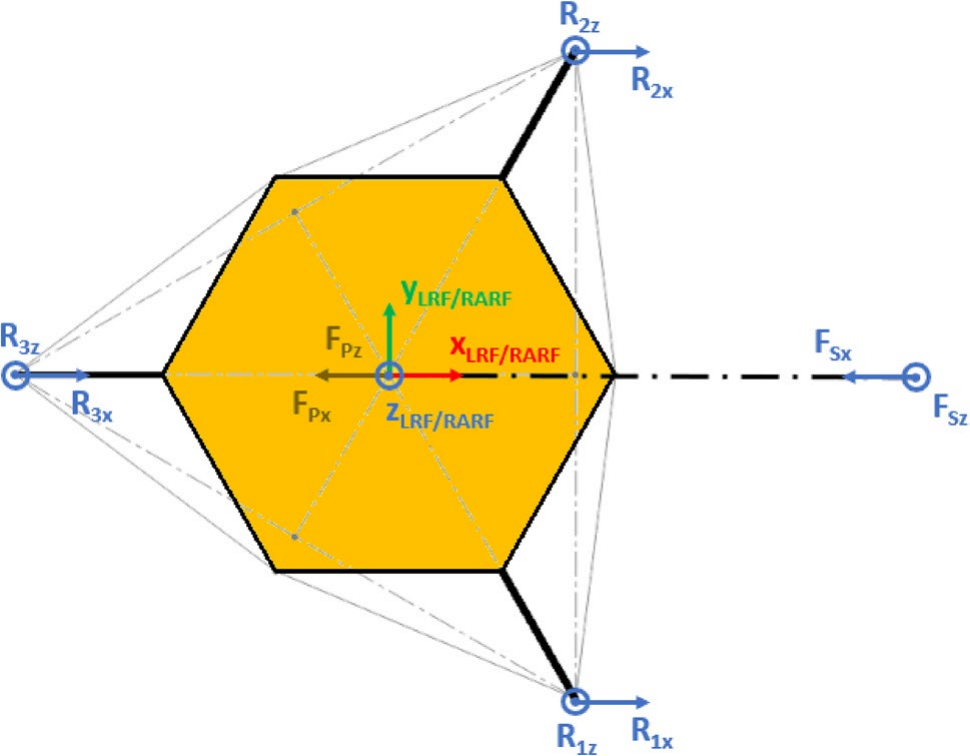
3-legged lander configuration for the Enceladus lander case study. The sampling spot is aligned along the *X* axis. Qualitative scheme, not to scale

**Fig. 20:**
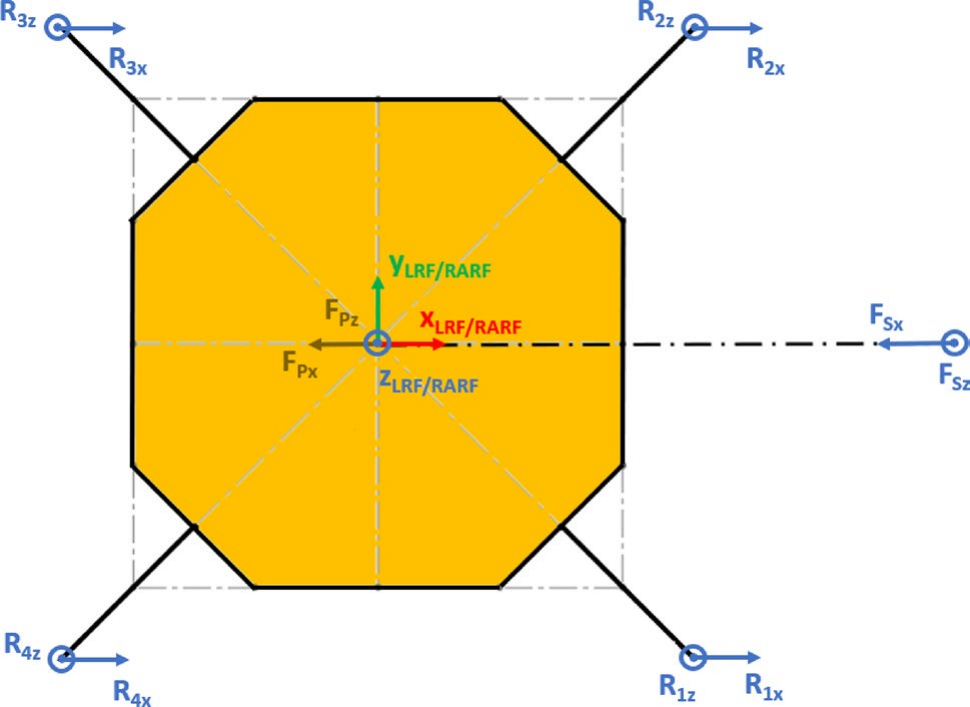
4-legged lander configuration for the Enceladus lander case study. The sampling spot is aligned along the *X* axis. Qualitative scheme, not to scale

The configuration under investigation is shown in Figs. 18, 19, 20. Such a configuration might be considered a worst-case scenario, since the lander is pushed downhill by the RA. The RARF was assumed being coincident with the LRF. The sampling force plane is aligned with the *X* axis, so the *Y* component of the sampling force is negligible. Moreover, since the sampling system considered is a scoop, the main component of the sampling force is along the *X* axis. This means that the goal is to avoid the lander to slide downhill, so the friction force along *X* axis is the driver to calculate the max sampling force within the defined margin.

The chosen values of the model parameters are shown in Table 5.

**Table 5:** Values of the model parameters for the Enceladus lander case study

Constant parameters	
**Environmental parameters**	**Value**
Gravitational acceleration $\left(g\right)$	0.113 m/s^2^
Slope of the ground about *X* axis $\left({\delta }_{g}\right)$	0°
Local slope of the ground about X axis at the sampling spot $\left({\delta }_{s}\right)$	0°
Local slope of the ground about Y axis at the sampling spot $\left({\gamma }_{s}\right)$	0°
**Physical and geometrical parameters of the lander**	
Length of the leg’s projection in the *XY* plane $\left({L}_{g}\right)$	0.5 m
Length of the body’s side $\left(D\right)$	1 m
[*X*, *Y*, *Z*] components of the ${C}_{G}$ position $\left({C}_{{G}_{x,y,z}}\right)$	(0, 0, 1) m
Margin on the reaction forces $\left({M}_{xy,z}\right)$	(1, 1)
**Physical and geometrical parameters of the sampling**	
*Y* component of the position of the sampling spot $\left({P}_{y}\right)$	0 m
Inclination of the sampling force with respect to the ground $\left(\beta \right)$	175°

**Variable parameters**	
**Environmental parameters**	**Range**
Slope of the ground about *Y* axis $\left({\gamma }_{g}\right)$	(0 ÷ − 20)°
Footpad-to-ground coefficient of friction $\left(\mu \right)$	0.5 ÷ 1
**Physical and geometrical parameters of the lander**	
Mass $\left(m\right)$	(300 ÷ 500) kg
**Physical and geometrical parameters of the sampling**	
*X* component of the position of the sampling spot $\left({P}_{x}\right)$	(2 ÷ 6) m

**Fig. 21:**
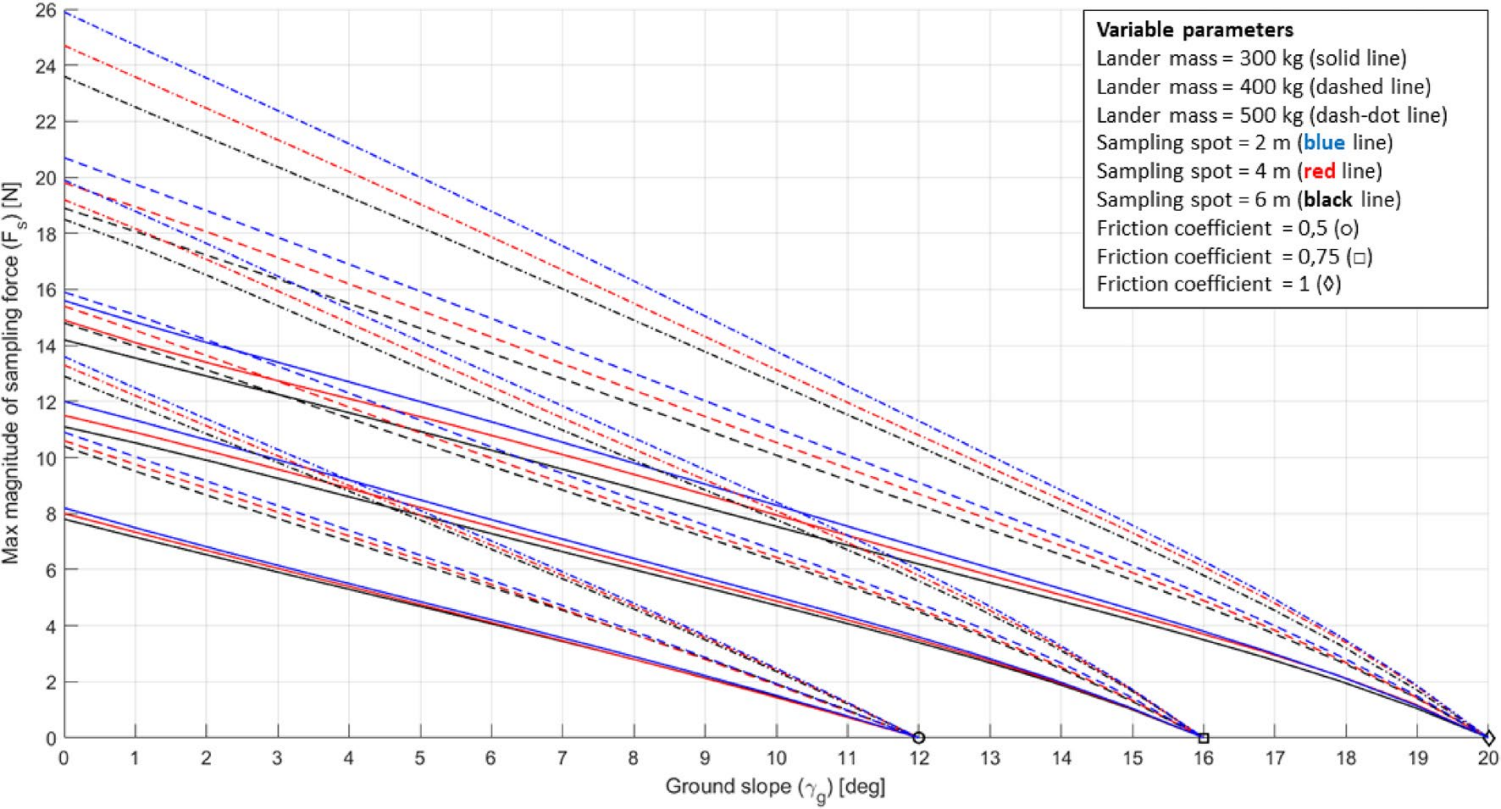
DE of the 3-legged lander for the Enceladus lander case study. The ground slope is reported in absolute value for convenience

**Fig. 22:**
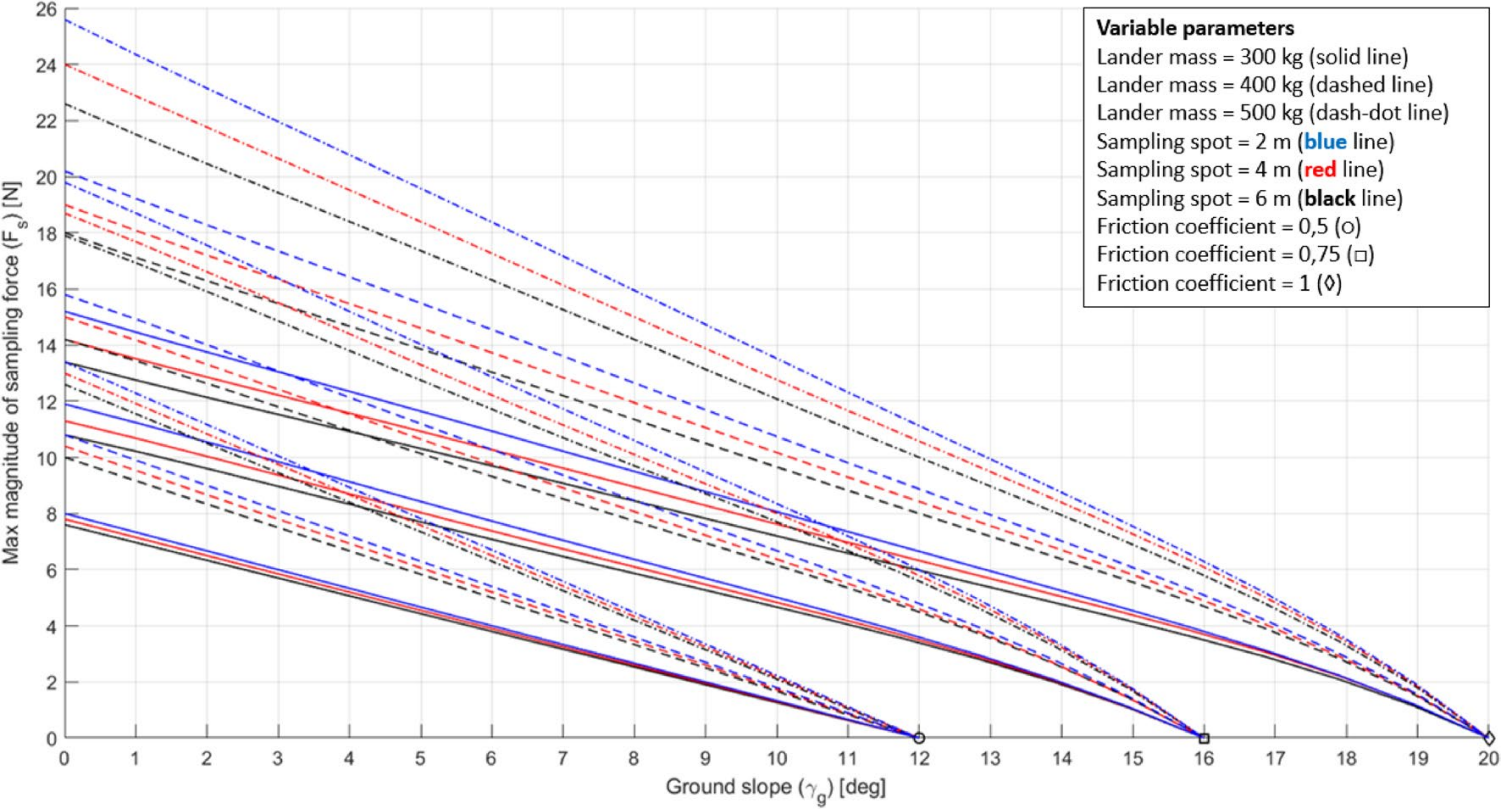
DE of the 4-legged lander for the Enceladus lander case study. The ground slope is reported in absolute value for convenience

The DEs obtained are shown in Figs. 21, 22.

DE plots were conceived to rapidly assess the high-level requirements of a sampling system. However, the benefits of using such a tool are much greater. Since the model used to build the DE considers the coupling among the sampling parameters, the lander parameters, and the environmental parameters, it is possible to use the plots to infer high-level requirements concerning other lander systems.

**Fig. 23:**
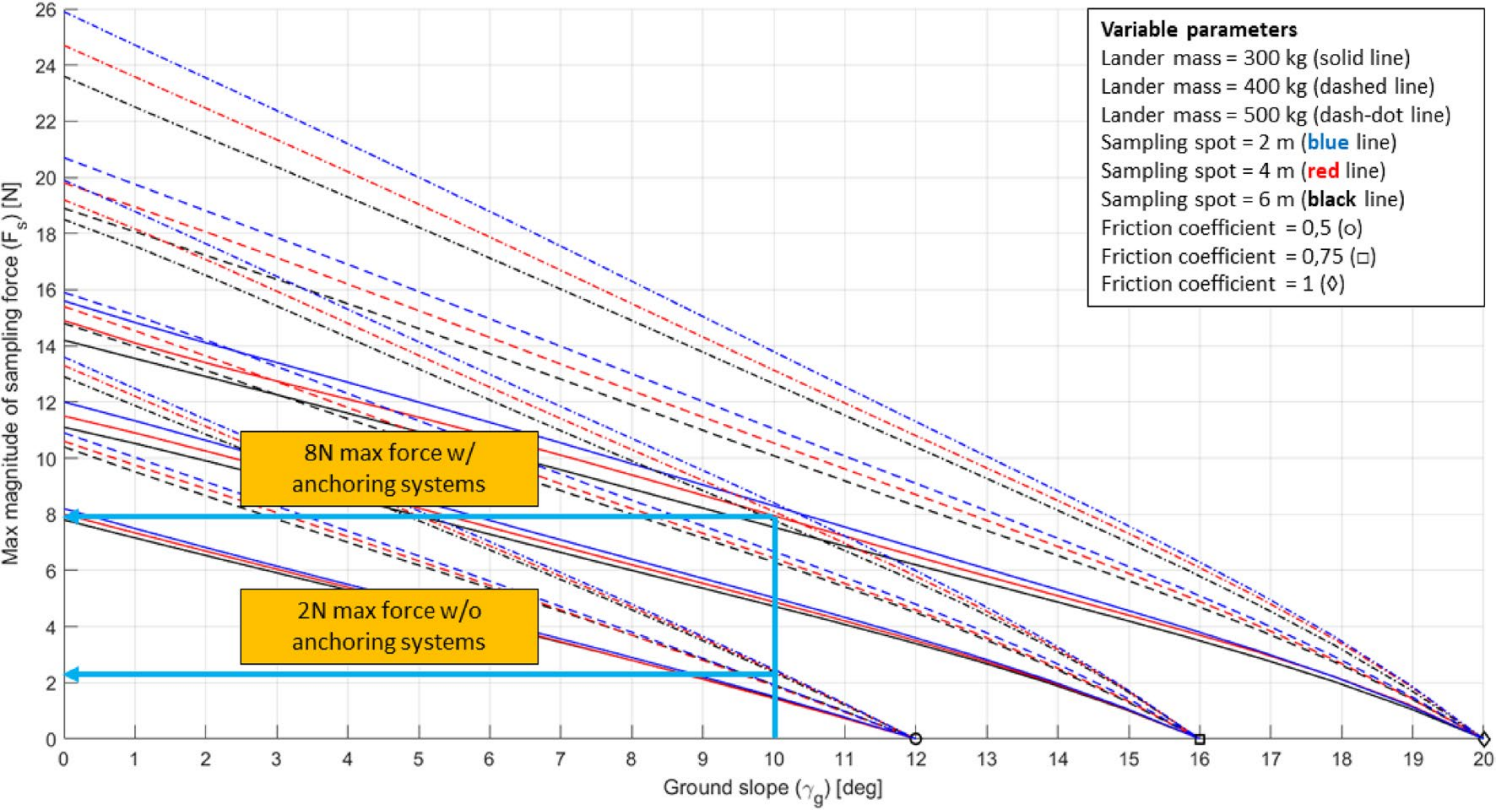
Use of the DE for general systems design. Example for the 3-legged lander configuration

From plots of Figs. 20, 21 it is possible to derive a requirement for the lander’s footpads, a critical system for lander’s stability. By assuming a lander’s mass of 500 kg and a 4-m-long RA, it is possible to derive that a coefficient of friction equal to 0.5 allows an incredibly low maximum ${F}_{s}$ (i.e. about 2 N). By increasing the coefficient of friction to 0.75 it is possible to sustain a maximum ${F}_{s}$ four times greater (i.e. about 8 N) (Fig. 23). A higher coefficient of friction can be achieved by adding small heated pins with the purpose of increasing resistance to lander’s footpad sliding. This turns into a requirement for both the sampling system (i.e. maximum allowed sampling force) and the lander system (i.e. footpad-to-ground coefficient of friction).

This example shows how powerful the tool is despite its simplicity, allowing for a broad and rapid overview of the design space, together with the most important parameters at play for systems design.

## Conclusions

Primary goals of robotic missions include sampling and sample collection for in-situ analysis or sample return to Earth. The analysis of robotic systems such as landers and rovers involved in sampling operations on planetary bodies is crucial to ensure mission success, since those operations generate forces that could affect the stability of the robotic system. The traditional approach for addressing such a problem involves iterative ad-hoc analyses to be repeated every time a new sampling tool, a new configuration of the whole robotic system or a new set of parameters defining the surrounding environment need to be evaluated. In order to make this process more time-effective and reliable, a systematic effort is required to provide a flexible and comprehensive tool to help defining the high-level requirements of a robotic system involved in sampling operations.

This paper presented MISTRAL, a novel tool conceived for trade space exploration during early conceptual and preliminary design phases, where a rapid and broad evaluation is required for a very high number of configurations and boundary conditions. The tool rapidly determines the preliminary DE of a sampling apparatus to guarantee the stability condition of the whole robotic system. The 3D analytical model implemented by the tool has shown the capability of reproducing several scenarios, being able to accept various input parameters, including the physical and geometrical characteristics of the robotic system, the properties related to the environment (i.e. gravity, physical and geometrical properties of the terrain) and the features related to the sampling system (i.e. geometry, applied forces). Despite its primary scope, the benefits of using MISTRAL are much greater. It was shown that DE plots can be used to infer high-level requirements concerning other lander’s systems, such as the RA, the footpads, etc. This comes directly from the ability of the model of considering the multidisciplinary coupling effects among the sampling parameters, the lander parameters, and the environmental parameters.

The tool has been applied to real mission data from Phoenix Mars lander. Moreover, MISTRAL has been adopted for the definition of the high-level requirements of the lander for a potential future mission to the surface of Saturn’s moon Enceladus, currently under investigation at NASA Jet Propulsion Laboratory. This case study was presented to demonstrate tool’s capabilities.

In conclusion, MISTRAL represents a comprehensive, versatile and powerful tool providing guidelines for cognizant decisions in the early and most crucial stages of the design of robotic systems involved in sampling operations on planetary bodies. Future developments include the possibility to analyze other lander configurations (i.e. body, legs and RA configuration). Moreover, quasi-static and dynamic analyses will be included providing the capability to analyze robotic systems equipped with dynamic sampling tools such as drills. Future versions of the tool will provide the possibility to consider the flexibility of the legs. The mass of moving parts of the RA and the tools attached at its tip (e.g. sampling tools) will be also included, alongside the analysis of rotational stability. Future activity will also include a tool validation campaign through experimental tests and/or simulation results. These improvements will pave the way of an extension of the tool to the analysis of rovers involved in sampling operations.

## Abbreviations

ISAD: Icy soil acquisition device
LRF: Lander’s reference frame
MISTRAL: MultIdisciplinary deSign tool for robotic sAmpLing
NASA: National aeronautics and space administration
RA: Robotic arm
RARF: Robotic arm reference frame
DE: Design envelope
DOF: Degree of freedom
3D: Three dimensional
